# Supplementary material for: MetaRibo-Seq measures translation in microbiomes
Source: Nat Commun. 2020 Jun 29;11:3268. doi: 10.1038/s41467-020-17081-z (PMC7324362; doi:10.1038/s41467-020-17081-z)
Supplement: Supplementary file 10 — Supplementary Data 7 [file 41467_2020_17081_MOESM10_ESM.zip › File2/Confidence_VeryHigh_Taxonomy/84427_out.krona.html]

Javascript must be enabled to view this page.

members
magnitude
magnitudeUnassigned
count
unassigned
taxon
rank

84427\_out

35

35
2
superkingdom

1
1239
phylum

186801
1
class

order
186802
1

31979
1
family

1485
1
genus

1
1896989

SRS054905\_contig\_number\_17825
species

976
34
phylum

200643
34
class

order

SRS1041139\_contig\_number\_contig-100\_11597.11598
1
171549
34

2005525
19
family

16
genus

SRS017821\_contig\_number\_contig-100\_80.107836SRS021948\_contig\_number\_contig-100\_891.207210SRS064557\_contig\_number\_19859SRS077024\_contig\_number\_12334SRS098571\_contig\_number\_contig-100\_21560.233545
195950
5

2
665949
species

SRS048870\_contig\_number\_18624SRS077127\_contig\_number\_16505

species

SRS052697\_contig\_number\_44792SRS142503\_contig\_number\_50009
2
1262979

2
2293248
species

SRS019397\_contig\_number\_9580SRS063190\_contig\_number\_14683

2293249
5

SRS018888\_contig\_number\_2943SRS019068\_contig\_number\_82906SRS048060\_contig\_number\_10481SRS104036\_contig\_number\_contig-100\_7112.46897SRS148784\_contig\_number\_contig-100\_21379.59747
species

genus
3
375288

1750560
1

SRS020869\_contig\_number\_contig-100\_5.217048
species

2292363
1
species

SRS055966\_contig\_number\_contig-100\_354.26973

1
328812

SRS063127\_contig\_number\_contig-100\_699.40229
species

2005519
14
family

genus
14
1348911


SRS013476\_contig\_number\_2021SRS013800\_contig\_number\_21099SRS015431\_contig\_number\_58304SRS016954\_contig\_number\_27078SRS017433\_contig\_number\_contig-100\_350.106117SRS019381\_contig\_number\_16690SRS024625\_contig\_number\_contig-100\_1384.116353SRS048164\_contig\_number\_contig-100\_4014.137166SRS077335\_contig\_number\_9502SRS1041039\_contig\_number\_14223SRS144506\_contig\_number\_55226SRS147346\_contig\_number\_43382SRS148196\_contig\_number\_42073SRS148424\_contig\_number\_19762
species
14
1099853
